# Conducting double-blind placebo-controlled clinical trials of transcranial alternating current stimulation (tACS)

**DOI:** 10.1038/s41398-021-01391-x

**Published:** 2021-05-12

**Authors:** Flavio Frohlich, Justin Riddle

**Affiliations:** 1 Department of Psychiatry, University of North Carolina at Chapel Hill, Chapel Hill, NC 27599 USA; 2 Carolina Center for Neurostimulation, University of North Carolina at Chapel Hill, Chapel Hill, NC 27599 USA; 3 Department of Neurology, University of North Carolina at Chapel Hill, Chapel Hill, NC 27599 USA; 4 Department of Cell Biology and Physiology, University of North Carolina at Chapel Hill, Chapel Hill, NC 27599 USA; 5 Department of Biomedical Engineering, University of North Carolina at Chapel Hill, Chapel Hill, NC 27599 USA; 6 Neuroscience Center, University of North Carolina at Chapel Hill, Chapel Hill, NC 27599 USA

**Keywords:** Physiology, Neuroscience

## Abstract

Many psychiatric and neurological illnesses can be conceptualized as oscillopathies defined as pathological changes in brain network oscillations. We previously proposed the application of rational design for the development of non-invasive brain stimulation for the modulation and restoration of cortical oscillations as a network therapeutic. Here, we show how transcranial alternating current stimulation (tACS), which applies a weak sine-wave electric current to the scalp, may serve as a therapeutic platform for the treatment of CNS illnesses. Recently, an initial series of double-blind, placebo-controlled treatment trials of tACS have been published. Here, we first map out the conceptual underpinnings of such trials with focus on target identification, engagement, and validation. Then, we discuss practical aspects that need to be considered for successful trial execution, with particular regards to ensuring successful study blind. Finally, we briefly review the few published double-blind tACS trials and conclude with a proposed roadmap to move the field forward with the goal of moving from pilot trials to convincing efficacy studies of tACS.

## Targeting network-level activity using tACS

**Fig. 1:**
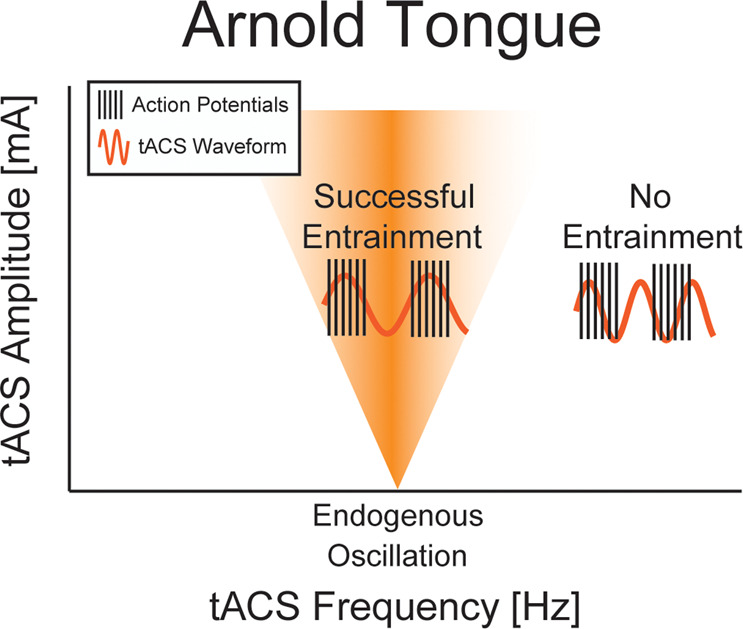
Successful neural entrainment from tACS is dependent on the Arnold Tongue. tACS entrains neural oscillations (represented as vertical lines symbolizing action potentials) by a mechanism referred to as the Arnold tongue, which references the inverted triangle shape of the parameter combinations (amplitude, frequency) that allow successful entrainment, i.e. synchronization of neural activity to the rhythmic stimulation waveform.The higher the stimulation amplitude, the more detuning (i.e., mismatch) of the stimulation frequency relative to the endogenous oscillation frequency is allowed to still achieve entrainment. Given the low amplitude of the perturbation to the neuronal membrane voltage by tACS, tuning of the stimulation waveform appears to be an important aspect of rational design of the stimulation waveform. Future tACS clinical trials should apply sine-wave stimulation waveforms that are matched to the endogenous frequency of the targeted oscillation determined by EEG. To what extent this principle predicts outlasting effects of tACS remains unclear given that there is no published preclinical model of these effects and that an attempt to investigate the role of entrainment in the outlasting effects was negative^39^.

Transcranial alternating current stimulation (tACS) is a form of non-invasive brain stimulation that applies low-amplitude rhythmic electrical current to the scalp for the modulation of cortical oscillations^1^. TACS has emerged in the wake of transcranial direct current stimulation (tDCS), which applies a constant current for the modulation of neuronal excitability and has been investigated as a potential therapeutic modality^2,3^. The rational for tACS is that the rhythmic (typically: sine-wave) waveform provides higher specificity by targeting oscillations in a specific frequency band in contrast to the more general modulation of excitability by tDCS^1,4^. After initial excitement about tACS, more critical voices started to dominate the conversation^5–7^. The main concern was about if and how the small perturbation of the membrane voltage can cause changes in neuronal oscillations. Specifically, the electric fields in cortex resulting from typical tACS paradigms is on the order of 0.5 V/m^8^, which results in a deflection of the somatic membrane voltage of around 0.5 mV, which is a fraction of the approximately 20 mV required for a neuron at its resting membrane voltage to fire an action potential. Do such minor perturbations to the membrane voltage by tACS indeed modulate network oscillations? Significant progress over the last 10 years has provided rather convincing answers to this question. First, experiments in acute cortical slices have demonstrated that comparable electric fields to the ones caused by tACS in cortex can modulate and entrain neural activity^9–12^. Second, computational modeling has served an important role in demonstrating that the inherently non-linear behavior of neurons around the threshold for action potential generation is maintained at the network level such that small perturbations can have a substantial impact on endogenous network dynamics^4,13^. Third, we now have a strong theoretical framework rooted in dynamics systems theory of how tACS entrains neuronal oscillations. It is known that periodic input to systems can entrain oscillators at very low amplitudes if the input is matched in frequency to the endogenous oscillation frequency of the oscillator. For increasing input amplitude, the range of frequencies around the endogenous frequency at which the input can entrain the oscillator expands, resulting in an inverted triangle of parameter combinations (stimulation amplitude and frequency) that successfully entrain an oscillator. This so-called Arnold tongue (Fig. 1) was first shown in computer simulations of tACS^4^ and was recently demonstrated in electrophysiological in vivo experiments^14^. More recent computational studies have confirmed the presence of the Arnold tongue for various models of neuronal networks and periodic stimulation paradigms^14–16^. Importantly, this mechanistic understanding of the importance of tuning the stimulation waveform to the endogenous oscillation provides an important design principle for future tACS clinical trials. Whenever feasible, the stimulation waveform should match the individual peak frequency of the stimulated research participant. In addition to this mechanistic work on tACS, recent studies in the non-human primate have confirmed successful entrainment of neuronal activity by tACS^17,18^.

Given this progress in our understanding of how tACS works, we argue tACS deserves a closer look as a potential circuit therapeutic for psychiatric and neurological illnesses. Electroencephalography (EEG) provides a library of activity signatures associated with psychiatric disease states that often point to specific pathological deficits in neuronal synchronization and oscillatory activity^19^. In other words, the rich literature of observational EEG studies provides candidate targets for the rational design of tACS interventions. The goal of this article is to advance double-blind placebo-controlled tACS trials by reviewing the theoretical foundation, sharing best practices for the design and implementation of tACS clinical trials, and reviewing the few published tACS clinical trials to develop a productive roadmap for moving forward.

## Rational design of clinical trials

One fallacy inherent to the use of non-invasive brain stimulation methodologies such as tACS is that the ease of applying stimulation can lead to an underestimation of the sophistication required in experimental design to be able to properly interpret the effects of stimulation. After acquiring a tACS device, applying stimulation is straightforward. As a result, it is tempting to dive head-first into running a tACS study without spending sufficient effort on the design of the study intervention and the trial itself. Broadly, the required steps (and goals of a trial) can be mapped onto three steps: target identification, target engagement, and target validation^20,21^.

### Target identification

As a first step, a network target in the form of a change in network oscillations, ideally detectable by scalp EEG, needs to be identified. The EEG literature should provide the required insights to identify a target. Examples are pathologically altered alpha oscillations as a marker of depression^22^ or impaired frontal theta oscillations during a cognitive control task as an indicator of treatment resistance in depression^23,24^. Ideally, the EEG literature should provide a correlation between a specific alteration in cortical oscillation and symptom severity. Of course, choosing a target based on such observational studies does not guarantee the correct identification of a target that responds to tACS. First, correlation is not the same as causation. Second, the EEG literature is quite diverse, and few truly consistent results exist. Part of the challenge is that the nosology of psychiatric disorders is highly contested and there are more recent efforts underway to reconceptualize psychiatric disorders in a more dimensional, rather than categorial, way^25,26^. The impact of dimensional approaches on tACS clinical trials remains to be seen, however, it does appear that the focus on neural processes as the organizing principles will facilitate the identification of more robust targets by accounting for individual differences. However, we are unaware of any published tACS clinical trial that has followed a dimensional approach for the definition of inclusion criteria. In view of this lack of well-established targets, all tACS clinical trials must include baseline EEG measurement and symptom assessments to demonstrate the presence of the stimulation target in the study sample. The target can be established through an exploratory analysis (correcting for multiple comparisons) or in a hypothesis-driven manner based on previous literature. One advantage of brain stimulation is that it can provide causal evidence for the role of specific oscillatory features in the disease process and symptoms. Therefore, a carefully designed tACS trial will provide causal evidence for a disease-brain connection. Importantly, however, the process of target identification should not be limited to a simplistic model of tACS boosting impaired oscillations. Rather, a more nuanced view is encouraged, since (repeated) stimulation may have opposing effects due to homeostatic processes. For example, TMS at 10 Hz has been proposed to reduce depression symptoms by re-normalizing (i.e., reducing) pathologically elevated left frontal alpha oscillations^22^. Perhaps the most direct evidence for such a mechanism of reducing a targeted oscillation is provided by the first clinical trial of tACS for major depressive disorder (MDD)^27^. It is also worth noting that stimulation at a frequency that is antagonistic to the frequency of interest can be used to indirectly reduce the strength of the target oscillation, such as applying stimulation in the gamma frequency band to reduce alpha oscillations^28^. In the subsequent sections on target engagement and target validation, we explain how clinical trials can either establish or exploit a causal relationship between neural activity and disease state.

### Target engagement

Once an oscillatory network target is identified, a stimulation paradigm is designed for successful target engagement. At the most basic level, the stimulation approach should include (1) positioning of the stimulation electrodes to deliver an electric field to the relevant brain areas and (2) a stimulation waveform that is tuned to the oscillatory target, following the above-outlined principle of the Arnold tongue. Although many tACS basic science studies seem to limit themselves to these considerations when designing the stimulation intervention, the process of designing the stimulation approach requires additional considerations. In terms of spatial targeting, electric field modeling can help to identify electrode positions that maximize the electric field in the target areas^29^. Colorful plots of electric field distributions should not be misunderstood as directly reflecting the maximal effect of stimulation on neuronal activity and oscillations. There are also important considerations such as the use of the “conventional” 2-electrode setup may not necessarily have the desired effect of enhancing oscillations since the neuronal populations underneath the two electrodes are modulated in their membrane voltage in opposite direction, and thus three or more electrode may be required to achieve the desired effects^30–32^. Importantly, if “conventional” electrode set-ups are used, three electrodes are required to achieve synchronization between two of the targeted areas by delivering in phase stimulation. More recently, electrode montages with a larger number of smaller electrodes have started to emerge^33–35^, which brings new opportunities of improved spatial targeting but also new considerations with regards to safety and mechanism of action^36^. Direct comparisons to more conventional (lower electrode count, typically with larger electrodes) are mostly missing (but see^37^) and of interest for future research. The motivation for more electrodes is straightforward in the sense that better spatial targeting can be achieved. What receives less attention but may be of equal importance is that (1) computer models suggest that the total number of neurons targeted matters^38^, (2) electrodes that are close to each other create electric fields that are less likely to penetrate into the brain due to increase shunting via the scalp, and (3) the chance of eventual successful translation into clinical workflows is reduced given the increased complexity associated with detailed electric field simulations and MR scans for the development of more complex electrode montages. In terms of temporal targeting, tuning the stimulation waveform in frequency is the first and most established step based on the recent demonstration of the Arnold tongue as the mechanism of action of tACS (Fig. 1). This principle can be broadly applied beyond the work targeting the individual alpha frequency, for example, a working memory task can be used to identify task-positive rhythms and their peak frequency such as the theta oscillation^35^. This can then be followed by consideration of features (such as non-sinusoidal waveform and phase-amplitude coupled signals) beyond the frequency of the targeted oscillation. Another parameter of the stimulation waveform that is worth considering is the stimulation phase. While the onset phase of the stimulation relative to the endogenous oscillation targeted by tACS seems to be an important parameter, a more careful examination of the response of oscillators to external periodic perturbations suggests a limited importance of phase, a view that has been now born out in computational^38^, preclinical^11^, and human studies^39,40^. Specifically, there are two lines of reasoning that support this latter view. First, the phase of endogenous neural oscillations is not stable in the absence of stimulation. Thus, whatever matching of phase that may be accomplished at the onsetof stimulation, is not guaranteed to persist beyond a few cycles of the oscillation. Second, more importantly, phase is a dynamical variable that converges on a stable equilibrium value (relative to the stimulation phase) in response to rhythmic periodic perturbations^41^. Thus, it appears, that for the duration of the typical tACS protocols phase at onset is less relevant since we expect a certain degree of entrainment during the stimulation. Tangentially, it is also worth noting that a recent invasive stimulation study has failed to provide evidence for successful synchronization of two areas when comparing anti-phase to in-phase stimulation^42^. Finally, and perhaps most importantly, we have a rapidly growing understanding of the instantaneous (“online”) effects of tACS on neuronal oscillations thanks to preclinical studies that examined the modulation of spike timing during tACS^9–11,43^. However, of higher relevance in the context of treatment clinical trials is of course the question about “outlasting” (“offline”) effects on network dynamics that persist beyond the application of tACS^39^. Very little is known about these effects and the underlying mechanism(s) of action, which leaves the parameter space in terms of stimulation amplitude, duration, frequency, and number of stimulation sessions under-constrained. Is daily stimulation required? Would paradigms that are more convenient for patients such as weekly stimulation work as well? Is there a gain in durability of effects by “booster” sessions after conclusion of the initial course of stimulation sessions? Until we have a mechanistic understanding of how tACS alters networks beyond the instantaneous effects, experimentation with different schedules is encouraged. Helpful insights may be derived from a recent observation that modulation of alpha oscillations by tACS may be dependent on brain-derived neurotrophic factor^44^, perhaps in a similar way to tDCS^45^. Similarly, there are individual case reports^46^ that suggest weekly administration of stimulation may be of clinical efficacy (and thus expected to cause lasting improvements to neuronal synchronization and oscillatory functional networks).

Overall, it is important to recognize that the “dose” of tACS spans a high-dimensional parameter space, which emphasizes the importance of mechanistic work to narrow the parameter space. For now, perhaps the most underappreciated but highly relevant aspect is that the effect of tACS heavily depends on the state of the brain, even basic differences such as the relative amplitude of the targeted oscillation during stimulation shapes the effect of tACS and other forms of rhythmic brain stimulation^43,47^. For example, pioneering work showed that the effect of tACS was limited to the “eyes-open” state^48^ when alpha oscillations are of low amplitude (in comparison to “eyes-closed” for which tACS did not have an effect). Computational and theoretical models help to understand these and related questions of state dependence^49^. More broadly spoken, it is also plausible that tACS has a fundamentally different effect in a patient population (with some kind of oscillopathy) in comparison to a healthy control population. Strikingly little is known to what extents such differences exist. Thus, including a healthy control population would further strengthen our mechanistic understanding of how tACS interacts with endogenous brain network dynamics.

In the context of tACS clinical trials, state-dependence has two important consequences. First, a consistent and well-documented state of the research participants during stimulation is of essence. Seemingly simple issues such as participants falling asleep during stimulation or participants engaging with their smartphones will lead to potentially quite different brain states and thus differential effects of stimulation on the targeted oscillatory activity. For example, drowsiness is associated with a slowing (lower peak frequency) and reduction in alpha oscillations^50^, which would cause a likely decrease in stimulation efficacy, which relies on matching the stimulation waveform to the frequency of the endogenous oscillations. Second, this state-dependence further explodes the parameter space of studies, which offers currently unexplored options such as combining tACS with cognitive-behavioral interventions for synergistic augmentation. In fact, it may be that the neural history, the circuit dynamics elicited prior to tACS, may modulate or enhance the response to tACS. For example, a psychotherapeutic intervention such as behavioral activation^51^ could be used as a pretreatment before application of tACS for the treatment of depression symptoms. Whatever stimulation paradigm is ultimately chosen for a given study, it is imperative to record neural oscillations before and after the intervention to delineate if target engagement was successful. To ensure that changes in the EEG are not the result of other factors, inclusion of a placebo group and an analysis of the difference between verum and placebo in the measures of target engagement is vital (see also below).

### Target validation

**Fig. 2:**
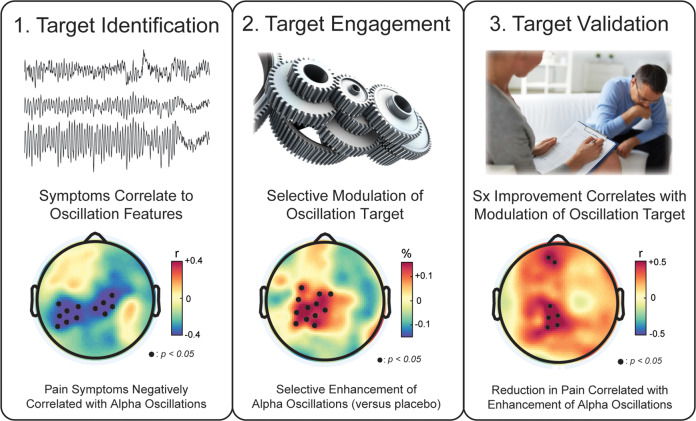
Rational design of clinical trials using tACS. Top: rational of a tACS intervention comprises **1** target identification (based on known correlations between EEG oscillation features and symptoms), **2** target engagement (demonstration of successful modulation of oscillatory network target), and **3** target validation (demonstration of clinical improvements correlating with change in oscillations by tACS). Bottom: results from a tACS study of target engagement and symptom modulation in patients with chronic low-back pain. In this case, the network oscillation target was pathologically reduced alpha oscillations (top-down inhibition) in the somatosensory-motor cortical pain network^60^.

Here, we define target validation as demonstration of the desired clinical or behavioral outcome by stimulation. Importantly, finding a significant difference between verum and placebo for a baseline normalized symptom score is an encouraging and important result. However, target validation ideally also includes more direct evidence that the successful target engagement (e.g., restoration of a specific neuronal oscillation) is indeed the cause for the clinical improvement. To date, the most compelling demonstration is the presence of a statistically significant correlation between individual differences in the effect of stimulation on EEG activation patterns and clinical symptoms scores. If such a correlation is present, the overall study provides convincing evidence for a causal role of the network target in the disease process and validates the investigated target for the treatment of the investigated illness (Fig. 2).

### The development of tACS as personalized medicine

The framework of rational design not only allows for the targeted development and investigation of novel tACS paradigms but also provides a blueprint for the further development of tACS in the framework of personalized medicine. Given the above-discussed importance of tuning the stimulation waveform to the characteristics of the endogenous network dynamics, adjustment of the stimulation paradigms to specific features in individual participants (such as the individual peak frequency of the targeted oscillation), represents a first step in this direction. Indeed, even in studies that did not perform such individualization, an analysis of the extent to which the stimulation frequency was out of tune for individual participants can be revealing when related to treatment outcome^52^. The next step is to utilize feedback stimulation, where the stimulation waveform is adjusted based on the measured activity signatures. This approach increases the match between the targeted network dynamics and the stimulation waveform, which can be expected to lead to improved target engagement. While fraught with technical challenges, an initial successful report of such feedback-tACS showed enhancement of sleep spindles and improvement in memory consolidation^53^, which appears to be a potentially attractive approach for treating cognitive deficits in people with schizophrenia^54^. Eventually, one can envision a future in which detailed network-based diagnostics of brain function can be used to develop targeted and individualized stimulation paradigms that work in synergy with other personalized treatments such as evidence-based psychotherapy tailored to individual clinical presentations and needs.

## Performing a high-quality tACS clinical trial

Once the framework of rational design is in place, focus should shift toward ensuring that the clinical trial is performed to the highest quality standards. While easy to understand the importance of this in theory, the reality on the ground is often that ad hoc decisions about seemingly minor details in study design and execution can have major unintended consequences that affect the interpretability of the resulting study. We here focus on aspects that directly relate (and are somewhat unique) to tACS clinical trials.

### Double-blind study designs

For all the obvious reasons, a double-blind trial design is a *sine-qua-non*. Given the magnitude of the placebo effect in (psychiatry) trials^55^, a control condition without the active ingredient that is masked from the participants and the study personnel is of critical importance. For tACS, “sham stimulation”, where all study manipulations are the same but the stimulator output is not turned on, represents an obvious, but insufficient, solution. TACS at amplitudes currently investigated is perceptible to most participants, albeit thresholds seem to vary quite a bit from participant to participant in our experience. Therefore, the use of an “active sham stimulation” as the placebo condition is highly recommended. In this approach, the first 60 s (arbitrary choice, some studies used up to 5 min depending on the population and the study specifics^56^) is the actual stimulation waveform which then is faded out (“ramp down”). The motivation for this method is that the sensations caused by activation of peripheral nerves are typically most strongly experienced at the beginning of the stimulation session likely due to heightened vigilance and because the peripheral nerves adapt to stimulation after a few minutes. Using such an active sham typically enables the successful performance of double-blind studies. While a recent review discusses different active placebo strategies for tDCS^57^, less has been written about tACS. For tACS, perhaps the largest concern is the presence of phosphenes, visual perception of “flickering lights” that is caused by activation of the optic nerve^58^. Phosphenes are more of an issue for more frontal electrode montages where higher current densities reach the retina via the eye socket. The perception of phosphenes during stimulation is modulated by the amount of visual processing. Therefore, stimulation in a dark room or with eyes closed is not recommended. In fact, the use of neutral visual input during stimulation is advisable. In trials from our group^27,32,59,60^, we project a slow-paced video of underwater scenes for the participant to watch to control for minimizing the perception of phosphenes and also for controlling the overall arousal of the participants to minimize state differences (at least due to visual input) between participants (and studies). It should be noted that these movie scenes are wide landscapes with minimal visual changes and are very different from the fast-paced editing styles used in film and television. No studies have addressed the specific role of watching such movie scenes during tACS and thus it is not clear to what extent one can generalize the effects of stimulation. However, one would expect that other relaxing (nature) movie scenes would equally work. Skin sensations that can be quite pronounced for tDCS are of less concern for tACS, likely since no long-term polarization of the tissue occurs. Several double-blind placebo-controlled clinical trials of tACS have queried for such sensations and have found no statistically significant difference between tACS and “active sham” ^27,59^. At least one group has advocated for the use of a topical anesthetic as a way to address skin sensations and to study the role of peripheral nerves by tACS^7^. Intriguingly, a later animal study has refuted the claims from this study^18^. While in principle an interesting idea, it is worth noting that the regulatory framework (at least in the United States) for a trial that includes off-label use of a prescription medication significantly increases complexity to the extent that it may stifle the field.

The inclusion of another active control condition is highly recommended. This ensures a tighter scientific contrast such that for example tACS at the frequency of the targeted oscillation outperforms tACS at a control frequency not involved in the targeted disease state. Of note, we recently contrasted 10 Hz-tACS with 40 Hz-tACS and an active placebo condition^27^. The treatment effect observed with 10 Hz-tACS was not found with 40 Hz-tACS, suggesting that the effect of tACS was frequency specific. However, our interpretation was complicated for the 40 Hz condition since participants were able to guess above chance that they received stimulation, likely due to increased phosphenes with 40 Hz. The frequency-dependence of phosphenes is thus another factor to consider when choosing active control conditions. Whatever exact setup is chosen, it is important to query both the research participants and the research staff if they believe active stimulation was administered and why. Asking for the reason is important to understand if unblinding occurred due to a difference in side effects such as phosphenes or are driven by subjective or objective improvements in symptoms.

There are additional practical factors to consider when conducting a double-blind placebo-controlled tACS clinical trial to achieve a successful study blind. There are both technical and human factors that require consideration. On the technical side, seemingly small design choices for the device can make a big difference. For example, devices with built-in batteries that display battery charge pose a risk for unblinding since the different charge level after stimulation versus placebo is marked and will unblind study personnel. Replaceable batteries do solve this problem but would require replacing the battery after each use, which would result in a high consumption of batteries, including the disposal of batteries with almost fully charge (used in placebo sessions). Economic and environmental factors make this choice less than ideal. Yet another approach is to use replaceable, rechargeable batteries. In this approach, the battery is swapped out after each stimulation session and the batteries are charged in bulk to avoid unblinding by the charger indicating an almost full battery after a placebo stimulation. Another technical factor to consider is how electrode impedance is measured and displayed during the sham stimulation. In a worst-case scenario, a device does not check impedance during sham stimulation. Stimulation cables and electrodes come loose more often than one would assume. Without impedance checks during sham stimulation, this will lead to an immediate unblinding of both patient and study personnel when the device pretends everything is fine despite a lead or electrode being clearly disconnected. Based on a recent review^57^ and our study of user manuals, only two devices currently measure impedance during sham stimulation (Neuroconn Plus, Neurocare Group, and XCSITE 100, Pulvinar Neuro, Durham, NC). Even a periodic impedance check during the sham stimulation mode carries a risk of accidental unblinding when impedance is displayed on the device in real time. Astute observers will recognize how the stimulation value is updated more often during verum than sham stimulation. Solutions for this include matching update intervals for impedance values for all stimulation conditions or masking the impedance value and simply displaying a symbol that indicates that impedance is low enough for stimulation.

In terms of human factors, the typical study population for pilot studies in academic settings brings its own risks of unblinding. On several occasions, we have had study participants who signed up for study participation and were incredibly well informed about tACS by reading the scientific literature. While such engagement may increase motivation to participate and to comply with study expectations, it also makes masking the placebo condition harder since the participants are aware of what stimulation effects such as phosphenes to expect. Study personnel can be accidently unblinded even if the stimulation device itself is specifically designed for double-blind studies. Studies that include EEG are also at higher risk for unblinding since an EEG running (even without recording) during stimulation will show the stimulation artefact. To address this, the sequence of stimulation and EEG recordings needs to be carefully designed to avoid such scenarios that can lead to accidental unblinding. Finally, good practices around who has access to the unblinding key are as vital as for any double-blind clinical trial. Details should be provided in the resulting publications since studies in this nascent field are often performed as small, investigator-initiated pilot studies where the reader cannot readily make the assumption that good practices have been implemented due to potential lack of experience or knowledge by the investigators.

### Interrupting and resuming stimulation

In contrast to applying 20 min of tACS in healthy participants, the reality of 40 min of tACS in (psychiatric) patient populations sometimes requires the interruption of stimulation for various reasons, e.g., an abrupt movement that causes a lead to disconnect. While not ideal, we believe that this disruption to continuous stimulation is acceptable as long as it is guaranteed that the correct amount of total stimulation per day is applied. Ideally, devices track the amount of stimulation performed and allow pausing the stimulation, followed by another ramp-in of the waveform for continuation of the stimulation for the remaining time. The same mechanism should be in place for stimulation interruption due to an impedance error. It is worth noting that there are no studies that explore what duration of interruption is acceptable without compromising successful target engagement or therapeutic efficacy. Pragmatically, given the difficulty of recruiting participants for such studies, we propose that cumulative interruptions less than 50% of the stimulation duration per session are acceptable, with the added recommendation of documenting such occurrences for future analyses pooled across studies.

### Stimulation validation

There is a common suspicion about tDCS studies that show inconsistent results, namely that confusing anode and cathode when preparing the stimulation could be the cause. While it is recommended to take such comments and stories with a grain of salt, there is some truth to them, namely that operator errors can very easily happen with tDCS and tACS. We speculate that one reason for that is that several of the leading companies are founded by engineers with no direct experience of human research and clinical trials. As a result, device interfaces are designed for engineers who of course are not the typical end users once the devices are sold to research groups in psychology, neuroscience, and medicine. Besides designing devices that are easy to use and do not require the user to think like an engineer, actual validation of stimulation is key to high-quality clinical trials. While traditional tACS does not have the problem with confusing the anode and cathode, there still can be issues such as application of the wrong waveform or failure of the stimulator to apply stimulation at all. For example, the Neuroconn Plus device can be purchased with an extra analog output that mirrors the applied current as voltage. Recording this signal provides documentation of the stimulation waveform for validation. Of note, this is not necessarily straightforward since the recording of this signal needs to be performed in a way that the study blind is maintained. Typically, this would include a computer with an analog-to-digital converter and custom-written code to capture the waveform without displaying it. Another, new approach is the recording of the actual waveform of both stimulation current and voltage (enables tracking of electrode impedance) into an encrypted file. This happens automatically in the background, does not require any additional hardware or software, and is guaranteed to maintain the study blind.

### Hurdles for participation in tACS clinical trials

TACS is extremely well tolerated and therefore drop out due to side effects is a very rare event. However, there are some other aspects to participating in a tACS clinical trial that require active management to maximize successful recruitment and minimize drop out. One aspect that has multiple implications is that attaching the electrodes requires that investigators have a prolonged presence in the personal space of the participant. In our experience, the reaction to this can be manifold, depending on the study population and individual preferences. Some participants enjoy the process and human contact from attaching electrodes and compare the experience to receiving a massage. Others take some time to get comfortable with the process. Often, such participants are focused on how their hair looks after stimulation, specifically the effect of the paste used to attach the electrodes and provide a low-impedance interface between electrodes and scalp (such as 10–20 paste). Training study coordinators to be extra mindful about personal space including specific strategies to minimize distress such as speaking at eye-level with the participant (requiring study personnel to sit down and explain steps while participant is seated), announcing individual steps and repeatedly asking for permission, regularly checking in with the participant about potential discomfort, and maintaining flow by engaging participants in small talk (if desired). It should not be lost on anyone that of course what may be well-intentioned small talk can be of (accidental) therapeutic benefit through mechanisms such as validation of emotional experience of participants, judgment-free acceptance of the perspective of the patient, and non-specific effects caused by positive social interactions. This is why an appropriate placebo condition in a double-blind design is essential. Training study personnel to avoid engagement with participant beyond small talk may be of help. It is worth noting that the participant and the study personnel spend significant amount of time together, which can lead to an increased level of comfort that may modulate the effect of stimulation but can also lead to disclosure of symptoms that have not been previously disclosed to the treating physician such as suicidal thoughts. A solid plan of action in case of such disclosures during study participation needs to be part of the standard operating procedures for any clinical trial, including tACS studies.

Time commitment for participating in a brain stimulation trial is a fundamental bottleneck. In contrast to a medication study that requires only a few minutes per day to take the study medication and document it, tACS (and other brain stimulation modalities) require a significant time commitment. While the ideal duration of a tACS session is unknown, the currently published trials used stimulation durations up to 40 min. Together with assessments, EEG measurements to document target engagement, and electrode application, a routine tACS study session can easily take 2 h per day. Thus, participating in a tACS clinical trial can collide with work, school, family, and social obligations. Potential mitigation strategies include offering stimulation sessions early in the morning or in the evening. This increased flexibility is greatly appreciated by interested patients but comes also with logistical challenges for the study team such as collisions with their life-work balance. In addition, brain activity measured by EEG exhibit circadian changes^61^ and thus, ideally, stimulation is applied in all participants in a similar time-window. Of note, however, it is unknown if the effect of tACS depends on the time of the day.

### Perceived conflicts of interest

The field of tDCS and tACS is nascent and many investigators (including the senior author, FF) work closely with companies that commercialize tDCS/tACS devices or have even started their own company (in the case of FF: Pulvinar Neuro LLC). While there is fundamentally nothing wrong with such industry interactions, there are important questions to consider to ensure the highest ethical standards and the quality of the research. First, it is important to ask if and how potential conflicts of interest are managed. Different universities may take different approaches (and overall rules seem to be stricter in the USA than in Europe, for example) leading to different levels of disclosures. First and foremost, we postulate the obvious, namely that complete and honest disclosure should become the norm. Papers should always include a complete statement that covers financial stakes, patents, paid and unpaid roles, and other facts that may be (mis-)understood as conflicts of interest. The same standards should also apply to editors and reviewers. Transparency is key. For example, who is the inventor of which patents related to tDCS and tACS? Sharing such information will ensure accountability and ultimately improve the quality standards of the research performed. For example, we conducted patent search (November 8, 2020, “transcranial AND alternating AND current AND stimulation AND tACS”) and found numerous patents related to tACS. Specifically, we found 143 patent filings in the USA, of which 54 are issued patents in the USA. Of note, the senior author of this review (F.F.) is listed on two hits in this search. We emphasize that filing patents are an important cornerstone of innovation. The research field of tACS would not be able to grow and develop without (mostly young and small companies) contributing to the development of the next generation of devices. We feel that radical transparency is the best way forward with regards to the management of conflicts of interest. Maintaining the integrity of the scientific enterprise should be of fundamental importance to all researchers in the field.

## Double-blind clinical trials of tACS

A PubMed search with the search string “double AND blind AND transcranial AND alternating AND current AND stimulation” revealed 76 references on November 1, 2020. We reviewed all 76 studies to identify studies that used tACS, employed a double-blind study design, and applied stimulation to patients (instead of healthy controls). We identified five tACS studies that used a double-blind placebo-controlled study design for the following patient populations: MDD^27^, auditory hallucinations in patients with schizophrenia^32,59^, chronic low-back pain^60^, substance use disorder (SUD)^62^, and Parkinson’s disease (PD)^63^. One study we do not discuss here used tACS in a population of people with attention deficit/hyperactivity disorder since it only focused on a single neurophysiological marker (P300 evoked response) and did not investigate potential therapeutic efficacy^64^. Another study that is closely related to the topic of interest but not a match for our criteria used the response to tACS in an attempt to investigate the progression from mild cognitive impairment to dementia^65^. We here briefly review the identified studies that provide a solid foundation for the next generation of tACS clinical trials and illustrate some of the key design considerations. Table 1 provides a synopsis of these trials.

**Table 1:** Summary of published double-blind placebo-controlled clinical trials of tACS.

Reference	Alexander et al.^27^	Ahn et al.^32^; Mellin et al.^59^	Ahn et al.^60^	Daughters et al.^62^	Del Felice et al.^63^
Patient population	Major depressive disorder (MDD)	Schizophrenia, schizoaffective disorder	Chronic low-back pain	Substance use disorder	Parkinson’s disease
Participants (*N*)	32	25	21	38	30
Study design	Placebo-controlled double-blind. Parallel group (10 Hz-tACS, 40 Hz-tACS, placebo-tACS)	Placebo-controlled double-blind. Parallel group (10 Hz-tACS, tDCS, placebo-tACS)	Placebo-controlled double-blind. Cross-over (10 Hz-tACS, placebo-tACS)	Placebo-controlled double-blind. Parallel group (10 Hz-tACS, 40 Hz-tACS, placebo-tACS)	Placebo-controlled double-bind, crossover (tACS, tRNS as placebo). Combined with physical rehabilitation.
Dose	1 mA zero-to-peak on each F3 and F4. Cz as return. 5 × 40 min.	1 mA zero-to-peak on each F3/Fp1 and T3/P3. Cz as return. 5 × 40 min	1 mA zero-to-peak on each F3 and F4. Pz as return. 1 × 40 min.	1 mA zero-to-peak on each F3 and F4. Cz as return. 1 × 40 min	1 mA to 2 mA (with DC offset) with individualized electrode montage (see text).10 × 30 min.
Primary Outcome Clinical	Null finding (pre-registered 4-week follow up), but positive on exploratory analysis (2-week follow up)	Null finding, but tACS numerically outperformed tDCS and placebo-tDCS	Positive	Positive (inhibitory control)	Null finding as defined (30% symptom reduction) but significant reduction of symptoms
Outcome Neurophysiology	Positive	Positive	Positive	None	Some evidence of target engagement
Pre-registration	NCT02339285	NCT02360228	NCT03243084	NCT03122587	NCT03221413

The major depressive disorder has been suggested to involved dysregulation of frontal alpha oscillations that can be targeted with non-invasive brain stimulation^22^. Synchronous stimulation of the left and right frontal cortex (EEG locations F3 and F4, with “return” electrode over Cz) was hypothesized to modulate left frontal alpha oscillations for 10 Hz-tACS and improve depression symptoms in patients with MDD. In a double-blind placebo-controlled trial^27^, we contrasted 10 Hz-tACS with 40 Hz-tACS and active sham tACS using a parallel group design. Stimulation amplitude was set to 2 mA peak-to-peak for each of the frontal electrodes (5 daily sessions of 40 min of stimulation). The primary clinical outcome was a significant difference in depression scores at the 4-week follow-up and the primary neurophysiological outcome was a statistically significant reduction of alpha oscillation on day 5 of stimulation. The primary clinical outcome was not met, however, there was a significant difference in responders (larger than 50% symptoms reduction) at the 2-week follow-up (exploratory analysis). The double-blind was successful for the 10 Hz but not the 40 Hz condition. The study was positive on the secondary EEG target engagement outcome (suppression of left frontal alpha oscillations). The main limitation of this study (besides the relative small sample size) is the lack of correlation between EEG changes and symptom improvement and the lingering questions about alpha asymmetry in depression^66^.

Auditory hallucinations can be conceptualized to arise from a lack of top-down control of excitability in auditory cortical areas. This study built on the initially promising results of tDCS for the treatment of auditory hallucinations^67^ with a study that contrasted tDCS with 10 Hz tACS and active sham tACS. Electrodes were positioned on F3/Fp1 and T3/P3 (with return on Cz) and stimulation amplitude was 1 mA zero-to-peak each for the F3/Fp1 and P3/P3 electrodes^59^. The primary clinical outcome was a significant difference in reduction in auditory hallucinations assessed by the Auditory Hallucination Rating Scale in favor of tACS, which was not met (albeit a numerical advantage of tACS over sham and tDCS was found, *N* = 22 participants). The primary neurophysiological outcome was an increase in left alpha oscillations, which was indeed found in the tACS group^32^. In addition, changes in left alpha oscillations significantly correlated with clinical improvement, providing direct support for alpha oscillations as treatment target.

Similarly, chronic pain can be conceptualized as pathological hyperexcitability in somatosensory(-motor) cortex. Given the inhibitory role of alpha oscillations, 10 Hz-tACS may increase pathologically decreased alpha oscillations and thereby improve symptoms. Indeed, a single session of 10 Hz-tACS versus active placebo increased alpha oscillations and caused an improvement in symptoms^60^. This cross-over study applied 1 mA zero-to-peak stimulation to both F3 and F4 (in phase), with the return electrode on Pz. Importantly, the study included target identification that showed how pain symptoms correlated with reduced alpha oscillations at baseline. The main limitation of this study is that only acute effects in response to a single stimulation session were assessed.

Impaired inhibitory control is a symptom of SUD^68^. Given the role of alpha oscillations in top-down inhibitory control^69^, tACS in the alpha frequency may increase inhibitory control in patients with SUD. To test this hypothesis 10 Hz-tACS was contrasted with 40 Hz-tACS and placebo tACS in a study sample of patients with SUD in an outpatient treatment facility^62^. In agreement with the study hypothesis, stimulation with 10 Hz-tACS but not 40 Hz-tACS or placebo-tACS showed a statistically significant increase in inhibitory control.

PD is associated with excessive beta oscillations in the basal ganglia and neocortex. A small, pilot clinical trial^63^ investigated the use of tACS to reduce pathologically elevated oscillations in patients with PD (10 daily sessions of 30 min). Stimulation frequency was set to 4 Hz for the case of “excess” beta oscillations and to 30 Hz in case of excess theta oscillations. The tACS waveform included a 1 mA DC offset and spanned 1–2 mA (peak-to-peak amplitude of 1 mA). Stimulation electrodes were positioned over the site of the highest peak oscillation power and the ipsilateral mastoid. The placebo condition employed transcranial random noise stimulation (tRNS). The study followed a cross-over design and included physical therapy immediately after each stimulation session. The primary outcome was a 30% reduction of UPDRS III off-medication score. The analysis included 14 participants and was negative on the primary outcome although a significant symptom reduction was found only for tACS and not for tRNS. The study interpretation is hampered by the small participant number, the diversity of different stimulation montages and frequencies (due to individualization), the potential efficacy of tRNS in modulating oscillations due to stochastic resonance, the lack of information of how double-blind stimulation was achieved, and the lack of a result report on ClinicalTrials.gov.

Together, these five clinical trials provide some evidence for the promise of modulation of cortical oscillations for the treatment of psychiatric and neurological disorders. Interestingly, most studies missed their primary symptom improvement outcome but showed promising target engagement results for the primary predefined EEG outcomes (and correlation with symptom improvement in some studies). Given the small sample sizes in these studies, the lack of statistically significant effects on symptom ratings is not surprising. Thus, overall, the interpretation of the results is limited by the small number of participants. The next studies should include larger sample sizes. Also, the choice of control conditions is worth further exploration in addition to more nuanced considerations and (reporting) of blinding procedures. It is worth emphasizing that including target engagement outcomes (EEG measurements of oscillatory network dynamics) have provided the most promising results in this first set of tACS studies and should thus remain the focus of the next tACS studies. This will avoid the pitfalls of tDCS trials where there are numerous clinical trials that do not include target engagement measures, which are ultimately needed to understand and interpret the disparate findings, including the recent larger negative trials of tDCS for the treatment of depression^70,71^.

## Outlook

One of the unique advantages of both tDCS and tACS is their extremely favorable safety profiles^72,73^. As a result, there has been a massive growth in tDCS and tACS studies. Perhaps, surprisingly, the number of placebo-controlled, double-blind tACS clinical trials is currently still very limited. The conceptualization of tACS as a potential treatment for oscillopathies is recent and we expect a strong growth in number of studies over the next few years. The goal of this article is to summarize the relevant information that we hope will increase motivation to perform high-quality clinical trials that follow a double-blind, placebo-controlled design. Defining the dose in tACS means picking a set of parameters from an even larger space than in the case of tDCS since frequency (and perhaps other waveform features, such as phase-amplitude coupling, rise-decay asymmetry, and periodic convolved with an aperiodic signal^78^) represents an additional dimension to consider. We thus argue that following rational design structured into target identification, engagement, and validation are essential to ensure that the field does not get stuck with few initial parameter choices that are then repeated across studies without significant innovation. Given that the mechanism of tACS is relatively well understood^74–76^, the field is ready for such a precision medicine approach. Building on the current findings, we expect that closed-loop tACS that can further increase target engagement through responsive stimulation^28,53,54,77^ will take the center stage in the next generation of studies. If the field focuses on trial quality (including larger sample sizes) and improved targeting, then the future will be bright for this low-cost and safe treatment modality.
